# Supplementary figures and images for: The gonococcal vaccine candidate antigen NGO1701 is a N. gonorrhoeae periplasmic copper storage protein
Source: PLoS Pathog. 2025 Oct 9;21(10):e1013559. doi: 10.1371/journal.ppat.1013559 (PMC12510493; doi:10.1371/journal.ppat.1013559)

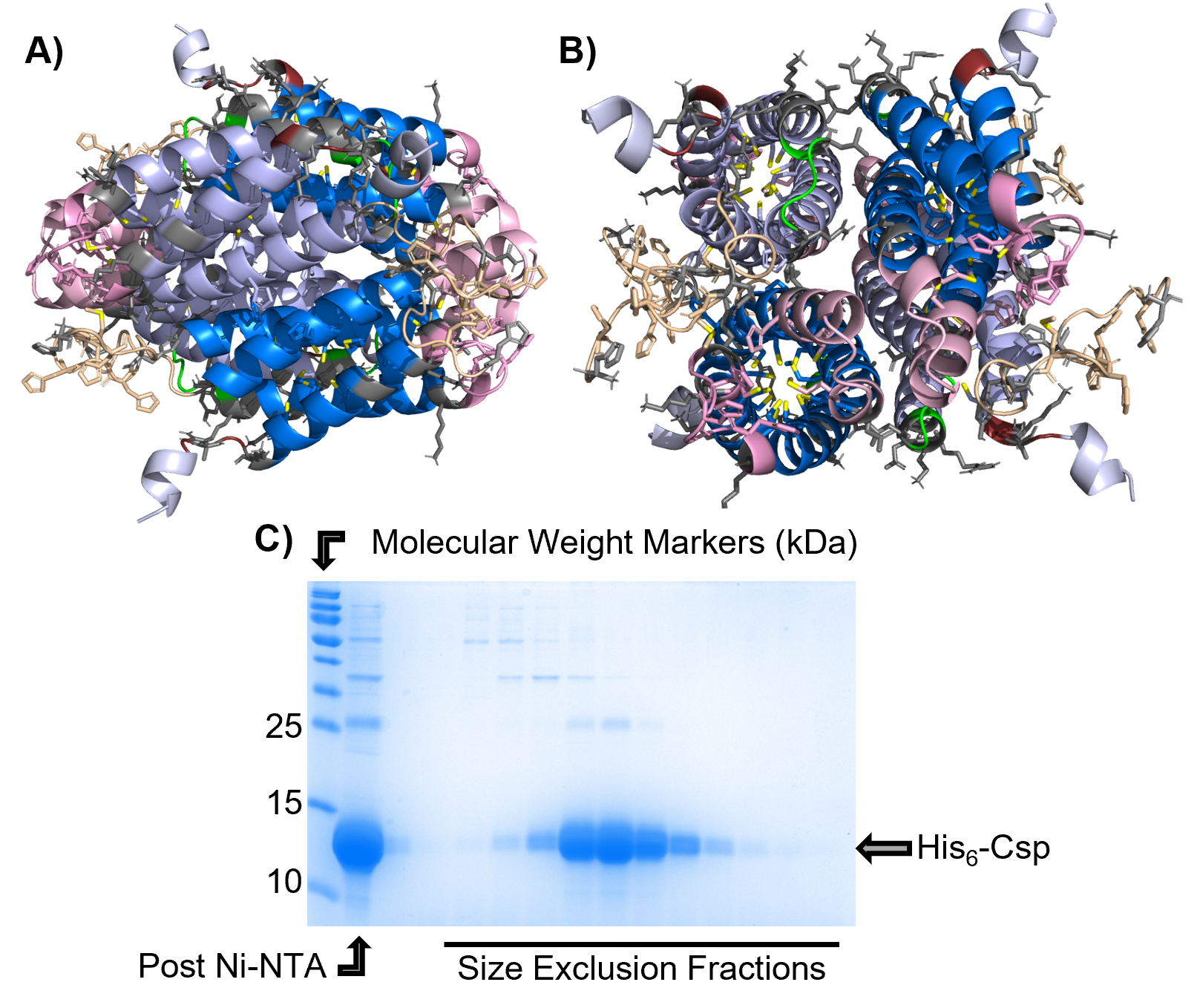

Supplement: S1 Fig — A) Side view of a cartoon model of Csp based on the Csp1 structural model created with GalaxyHomomer (one pair of symmetry-related monomers shown in dark blue and the other pair in light blue). B) Front view (90° rotation relative to A). The colored regions illustrate the predicted linear epitopes (LE) 1–5 and conformational epitopes (CE) 1–3 [11], located on the outside of the tetrameric structure, validating the model and confirming this tetrameric form as the likely structure in solution. C) SDS-PAGE and Coomassie staining of purified recombinant Csp fractions from size exclusion chromatography following Ni-NTA column chromatography. Fractions were resolved on a 16% polyacrylamide gel. (TIF) [file ppat.1013559.s001.tif]

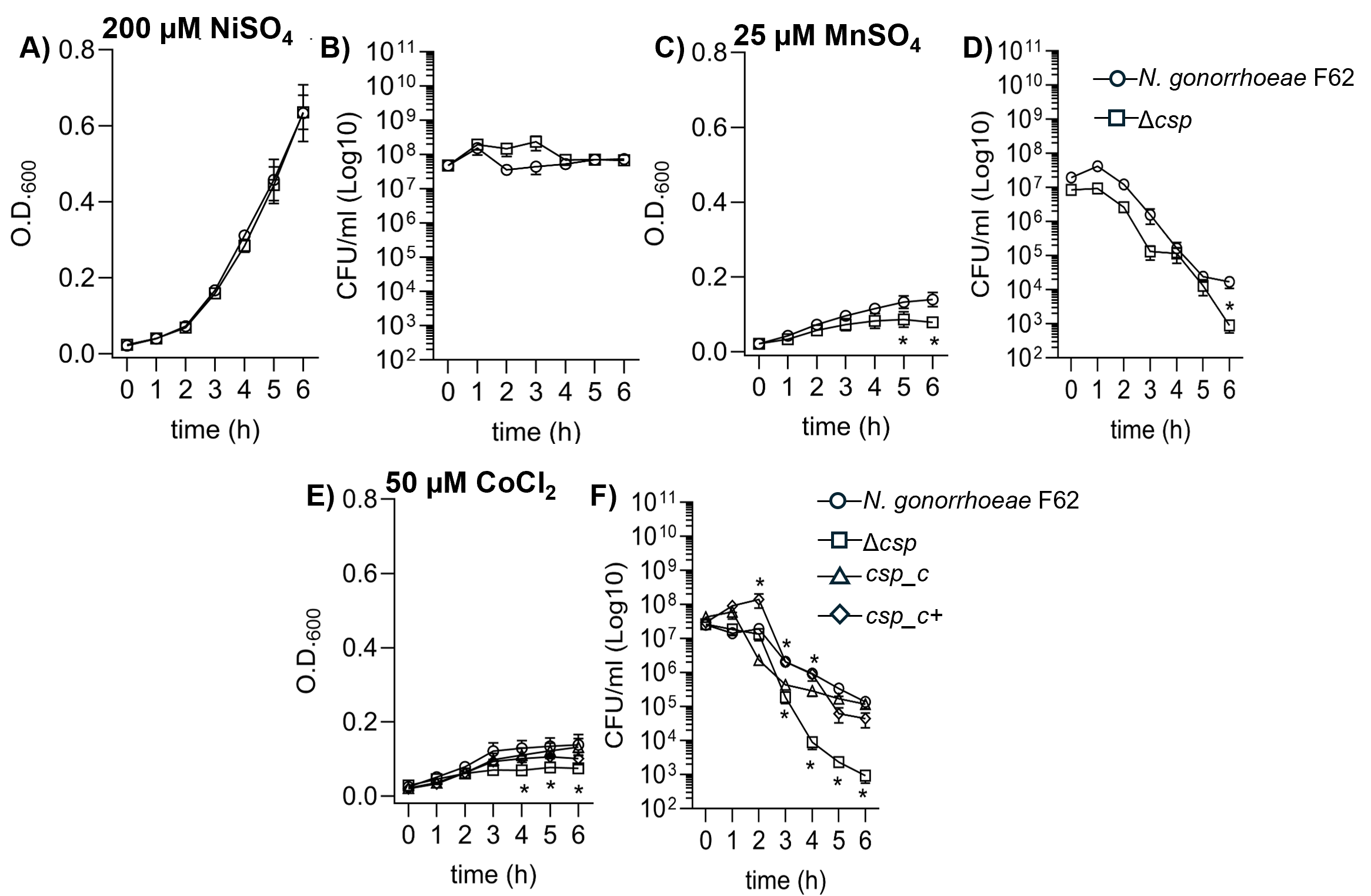

Supplement: S2 Fig — OD600 values (average ± SEM) from three independent growth curves in the presence of A) 200 µM nickel sulfate (NiSO4), C) 25 µM manganese sulfate (MnSO4) or E) 50 µM cobalt chloride (CoCl2). * p ≤ 0.05 vs no treatment by 2-way ANOVA with Dunnett’s multiple comparisons test. B-D-F) CFUs/ml (average ± SEM) as above. Statistical significance was determined by multiple Mann-Whitney test with Holm-Sidak correction set for p = 0.05 vs no treatment for each strain, indicated by *. N. gonorrhoeae F62 wildtype (circles), Δcsp (squares), csp_c (triangles) and csp_c+ (diamonds). (TIF) [file ppat.1013559.s002.tif]

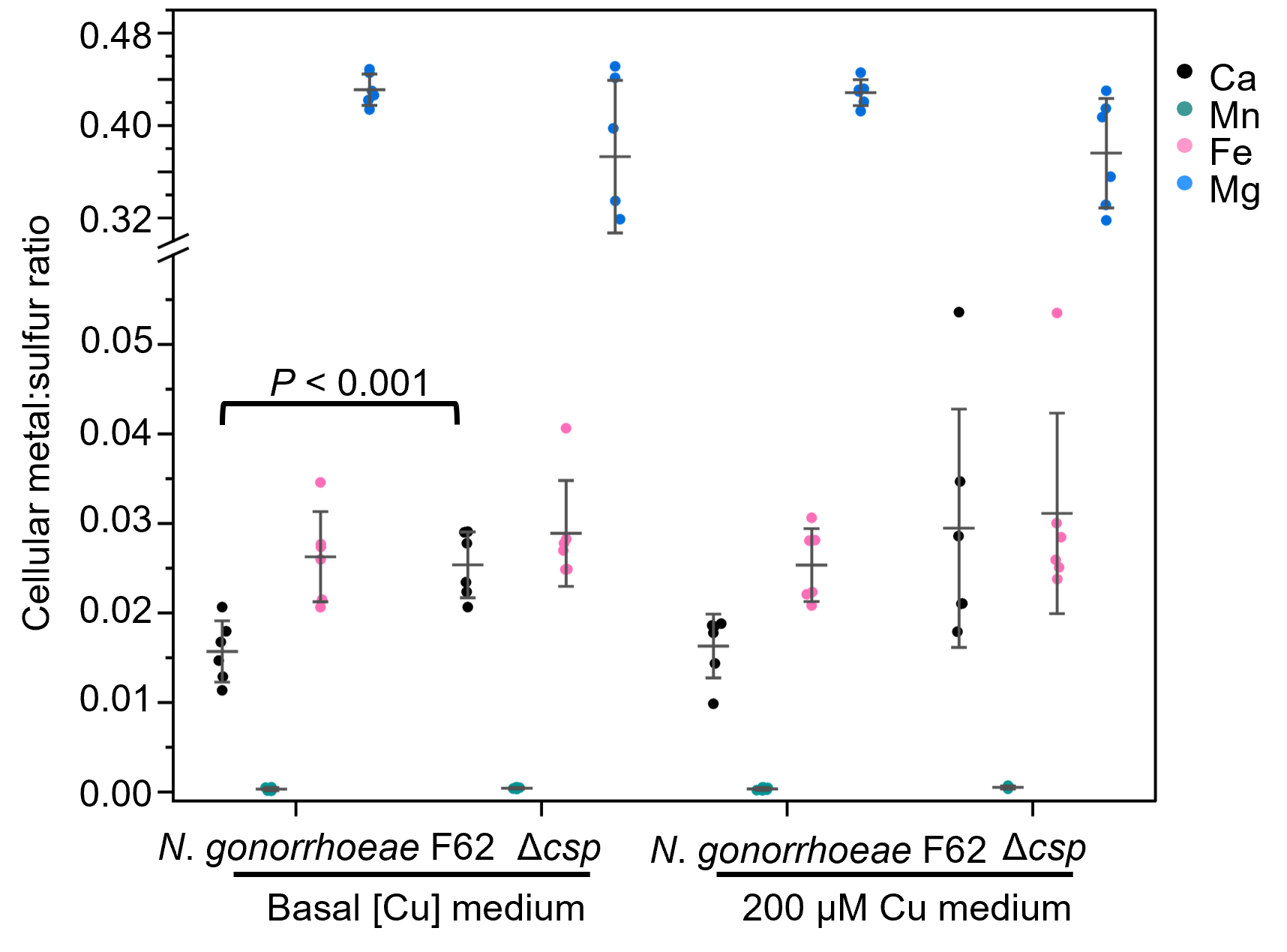

Supplement: S3 Fig — After removing residual surface-adsorbed metal, bacterial pellets were digested and analyzed for Ca (black), Mn (teal), Fe (pink), Mg (blue) and S (sulfur) by ICP-OES. Metal content was normalized to S content to account for differences in biomass. P represents the results of an unpaired t test. (TIF) [file ppat.1013559.s003.tif]

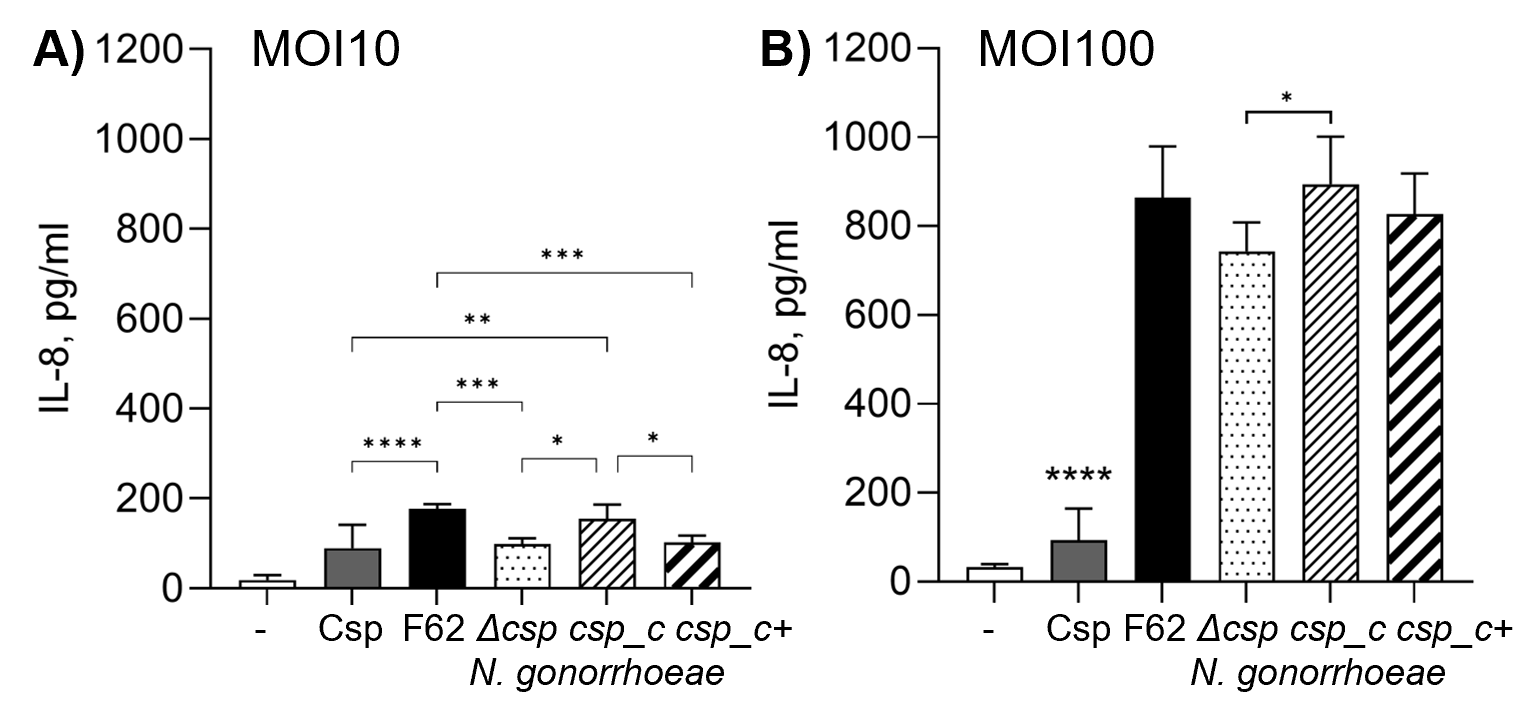

Supplement: S4 Fig — Cells were incubated with purified Csp (10 μg/ml) (gray bars), N. gonorrhoeae F62 wildtype (black bars), Δcsp (dotted bars), csp_c (thin striped bars) or csp_c+ (thick striped bars) at A) MOI 10 or B) MOI 100 for 18 h. IL-8 secretion was measured in cell culture supernatants by ELISA and expressed as pg/ml ± SEM from triplicate wells. * p < 0.05, **, p < 0.005, ***, p < 0.0005 and ****, p < 0.0001 by Ordinary one way ANOVA with Tukey’s multiple comparison test. (TIF) [file ppat.1013559.s004.tif]
