# Supplementary material for: The gonococcal vaccine candidate antigen NGO1701 is a N. gonorrhoeae periplasmic copper storage protein
Source: PLoS Pathog. 2025 Oct 9;21(10):e1013559. doi: 10.1371/journal.ppat.1013559 (PMC12510493; doi:10.1371/journal.ppat.1013559)
Supplement: S1 Table — (DOCX) [file ppat.1013559.s005.docx]

| **S1 Table. List of primers and sequences** | |
| --- | --- |
| **Primer name** | **Sequence (5’-3’)** |
| *ngo1701* upstream_F | GGCCAAAGGCATGAAGGGAATAAAGGG |
| *ngo1701* upstream_R | CGGATGGAACCAGGCTGCATATCGTGATGATGGTGGTAG |
| *ngo1701* downstream_F | GCAAAGAAGCTTCGAGTCCTGCCTCGACTGTATCAAAGAATG |
| *ngo1701* downstream_R | TTGCCCGATGTGGCGATTGCTAAAG |
| pGCC4 Kan_F | CATTTCGAATTCCAGAGTCCCGCTCAGAAGAAC |
| pGCC4 Kan_R | ATGGACAAGCTTCGAACCGGAATTGCCAGCTGG |
| *ngo1701*_F check | TGCAGCAGATGATGAAGATGTTC |
| pGCC4 mid_Kan | TACGCTTGATCCGGCTACCT |
| *lctP*_F | CTGTCCGTGACCCTGATTCTGGC |
| *acpC*_R | GATGCCGAGGTTGACTTTTT |
| *lctP*_F and *lacIq*_R | CCACCCTGAATTGACTCTCTTCCGG |
